# The Teeth and Mouth of Idiots

**Published:** 1872-05

**Authors:** 


### ARTICLE IX.

### *The Teeth and Mouth in Idiots.*

In a previous number we gave an abstract of the observations of Dr. Langdon Down in reference to certain characteristics of the mouth and teeth of congenital idiots. Dr. J. W. White, editor of the *Dental Cosmos*, has been making some observations in the same direction, and thus records in that journal the result:

"The views expressed by Dr. Down, so far as they related to the narrow arch and vaulted palate as associated with mental defect of congenital origin, are in exact accordance with opinions which the writer had long entertained and frequently expressed—opinions originating, probably, from observations of the mouths of a family most of whose members were considered as lacking in mental power; one of whom—the most markedly deficient—presenting in extreme the peculiar mouth under consideration, which characterized to a greater or less extent the entire family.

"To investigate the facts, more especially in reference to the other deviations from normal conditions, we recently sought and obtained from the gentlemanly superintendent, (Dr. J. N. Kerlin) of the 'Pennsylvania Training School for Feeble-minded Children,' an invitation to spend a day at that institution. In company with an esteemed dental friend, who took a deep interest in the problems presented, but who had formed the opinion that the characteristic mouth of idiots was abnormally flat and broad, we devoted the day to an examination of the dental and other peculiarities of the inmates, numbering about one hundred and eighty.

"The first three cases which presented were, by a singular coincidence, so strongly confirmative of Dr. Down's position in reference to the narrow and deep arch, that there was corresponding assurance on the part of one of the examiners, and distrust on the part of the other, as to the correctness of previously formed opinions. The next case was the exact reverse, a broad, flat mouth; the evidence being strongly in favor of the congenital origin of the mental defect, which was decidedly pronounced. To these succeeded a series of cases which were absolutely free from any noteworthy peculiarity of the oral cavity. Among the balance we found three more marked cases of narrow and vaulting arches; but many where the deviation, if any, from normal condition was in the direction of breadth and flatness rather than the reverse. There were two cases of V-shaped upper maxillary; two of disproportionate size of lower jaw; several of *unus-*

*ually* broad and flat arches; one case of hanging and one of cleft palate; and several cases of incomplete dentition—a missing lateral or canine. We could not determine any special deviation as to time or sequence in dentition, and were forced to the conclusion that, considering the inattention to their teeth, as to everything else connected with the care of their persons, the teeth stood the test of neglect remarkably well.

"In the mouths of some of the inmates—especially those whose mental development was little above zero—there were teeth which, for size, regularity, density, and perfection of form would answer as models. There were also teeth which were faulty in every respect and relation; but on the whole, we had to admit they were about an average lot—neither better nor worse than those of the same number of similarly neglected people of ordinary intelligence.

"There is one fact apparent to the most superficial observer—the rapidity with which these poor creatures grow physiologically old. The evidence of senility are noticeable in every organ and function; in premature baldness and gray hair; in dulness of hearing and dimness of vision; in the wrinkled skin; the tottering step; the wasted limbs; the receding gums, and absorbed alveolus. So that, in several cases, the man of thirty, by the record, seemed to be on the other side of sixty. Even in these cases, the teeth which were ready to drop from their sockets were not unfrequently sound in their structure.

"Subsequent examination of the mouths of a number, believed to be congenital idiots, has resulted in finding three more cases of the high and narrow palatal arch; but these were counter-balanced by other cases where the development was exactly the opposite, and by others where there was no discoverable abnormity; while several marked instances of the mouth described by Dr. Down have been found associated with intelligence of a decided character; one instance, in particular, of excessive height and extreme narrowness—more marked than any we found among the feeble-minded

—in the case of a very intelligent and successful professional man.

"Notwithstanding these apparent contradictions, it seems reasonable that there is a correlation between physical and mental organizations; and the deductions of one who has had such ample opportunities and so extensive an experience as Dr. Down are not to be set aside by the observation of a day, even if no solution of the problem presented; but it is presumable that the class of imbeciles which came under Dr. Down's inspection differs, as a rule, from the majority of those who are to be found here, in such an institution as the one mentioned.

"We suggest as a reasonable explanation the probability that the cases of which our opportunities allowed an examination were chiefly the result of accidental, individual causes occurring during gestation, or inhering only in the immediate progenitor—perhaps in an exceptional condition at the time of impregnation or conception, such as the perversion of function produced by inebriation or other perturbating influences; while Dr. Down's cases were probably examples of continuous enervating and degenerating influences of inter-marriage and other depressing causes connected with the life of the aristocracy. These causes being cumulative through successive generations, would doubtless produce a different style of abnormity from that which is the result of individual and accidental causes. That a law of correlation between progressive mental and physical development or deterioration exists, there can be no doubt, nor that it can be traced, if we 'observingly distil it out'; but problems like these are not definitively settled except by the concurrent testimony of various observers."—*Med. Times.*

